# Retraction: A Functional Nuclear Epidermal Growth Factor Receptor, Src and Stat3 Heteromeric Complex in Pancreatic Cancer Cells

**DOI:** 10.1371/journal.pone.0212884

**Published:** 2019-02-20

**Authors:** The PLOS ONE Editors

Following publication of this work [1], the following concerns were raised:

- Fig 1A (ii) Stat3 panel appears to similar to the Input Src panel when horizontally flipped, and to the IP: Stat3/Src blot panel in Fig 1C (i) when flipped vertically
- Fig 2A (i) Input: EGFR panel appears similar to Fig 1A (i) Input: EGFR panel
- Fig 3A the Stat3 blots for input samples appear similar in (i) and (ii)
- Fig 3C the EGFR and Src blots shown for input samples appear to be similar in (i) and (ii)
- Fig 3B EGFR lanes 1–3 appear similar to lanes 7–9
- Fig 6B (i) Src panel appears similar to Fig 3A (i) Src panel
- Fig 6B (ii), in the β-actin panel there appears to be a vertical discontinuity between lanes 3 and 4, and lane 4 appears to have a different background as compared to other lanes in the panel

The authors have noted that all figure panels represent independent experiments. However, they have explained that in Fig 3A, the same Stat3 input panel was presented in panels (i) and (ii) because the same input control data was relevant to both Stat3 immunoprecipitation experiments. The immunoprecipitation experiments shown in panels (i) and (ii) used the same amount of the same samples. In Fig 6B (ii), authors commented that differences in background could be attributed to lower levels of protein in lane 4 compared to lane 3.

While the authors stand by the validity of the experimental data and the conclusions reached in the study, owing to the time passed since the experiments were completed they were unable to provide any of the original images or raw underlying data in the article. Hence, we have been unable to resolve the above concerns.

In light of the image concerns and the unavailability of primary data, the *PLOS ONE* Editors retract this article.
