# Correction: Retraction: A Functional Nuclear Epidermal Growth Factor Receptor, Src and Stat3 Heteromeric Complex in Pancreatic Cancer Cells

**DOI:** 10.1371/journal.pone.0216490

**Published:** 2019-04-30

**Authors:** The PLOS ONE Staff

There is an error in the Notice of Retraction published on February 20, 2019 [1]. The notice states that the authors were unable to provide any of the original images or raw underlying data in the article [2]. However, the data underlying Fig 4 is available and was provided to the journal by Dr. Qun Huo.
